# ASO Author Reflections: Impact of Resection Volume on Risk of Local Recurrence—The OPBC-01/iTOP2 Study

**DOI:** 10.1245/s10434-021-10862-w

**Published:** 2021-09-28

**Authors:** Giacomo Montagna, Walter P. Weber, Angelika Geroldinger, Florian Fitzal

**Affiliations:** 1 Breast Service, Department of Surgery, Memorial Sloan Kettering Cancer Center, New York, NY USA; 2 Breast Center, University Hospital of Basel, Basel, Switzerland; 3 University of Basel, Basel, Switzerland; 4 Center for Medical Statistics, Informatics and Intelligent Systems, Medical University of Vienna, Vienna, Austria; 5 Department of General Surgery and Breast Health Center, Medical University Vienna, Vienna, Austria

## Past

Oncoplastic breast conservation (OBC) techniques can be divided into low- to intermediate-volume (Clough level I, Tübingen 3,4) and large-volume (Clough level II, Tübingen 5,6) resections with removal of less or more than 20% of the breast tissue, respectively.^1,2^ The use of these techniques has become increasingly popular over the past 2 decades as they merge the curative intent of oncological surgery with plastic surgery techniques in an attempt to resect larger and/or multicentric tumors, reducing the necessity to perform mastectomies.^3^ However, most of the evidence evaluating the benefit of OBC is based on small single-center studies, limiting its applicability and generalizability.^4^ In this global retrospective study, we sought to determine whether OBC with large resection volumes improves local recurrence rates compared with conventional breast conservation or low-volume oncoplastic procedures in women with high-risk breast cancer.

## Present

We included 3177 women from 15 institutions in 8 countries within the Oncoplastic Breast Consortium (OPBC) network between January 2010 and December 2013. Of these women, 30% were treated with OBC (OBC Level I, *n* = 663; OBC Level II, *n* = 297) and the remaining 70% were treated with conventional breast-conserving surgery (BCS). The great majority of patients (92.3%) had invasive cancer, while the remaining patients had high-grade ductal carcinoma in situ (DCIS). Among patients with invasive cancer, 41% were luminal B-like, 27% were human epidermal growth factor receptor 2-positive (HER2+), and 21% were triple negative.

Patients treated with BCS/OBC Level I had significantly smaller tumors and smaller resection margins compared with patients treated with OBC Level II (pT1: 50% vs. 37%, *p* = 0.002; proportion of patients with a margin <1 mm: 17% vs. 6%, *p* < 0.001). There were significantly more re-excisions due to R1 (‘ink on tumor’) in the BCS/OBC Level I group compared with the OBC Level II group (11% vs. 7%; *p* = 0.049). At a median follow-up of 74.5 months, there was no difference in local, regional, and distant recurrence-free and overall survival between the two groups.^5^

## Future

Oncoplastic Level II resections increase margin width and decrease the number of re-excisions due to positive margins, but are not associated with lower local recurrence rates. The results of this study reinforce the concept that in invasive cancer, margins larger than ‘no ink on tumor’ do not improve local control.
